# Improved Photopyroelectric (PPE) Configuration for Thermal Effusivity Investigations of Porous Solids

**DOI:** 10.3390/ma16072880

**Published:** 2023-04-04

**Authors:** Carmen Tripon, Mohanachandran Nair Sindhu Swapna, Nicoleta Cobirzan, Dorota Korte, Robert Gutt, Marcel Bojan, Mladen Franko, Dorin Dadarlat

**Affiliations:** 1 National R&D Institute for Isotopic and Molecular Technologies, Donat 65-103, 400293 Cluj-Napoca, Romania; robert.gutt@itim-cj.ro (R.G.); marcel.bojan@itim-cj.ro (M.B.); 2 Laboratory for Environmental and Life Science, University of Nova Gorica, Vipavska 13, SI-5000 Nova Gorica, Slovenia; swapna.nair@ung.si (M.N.S.S.); dorota.korte@ung.si (D.K.); mladen.franko@ung.si (M.F.); 3 Faculty of Civil Engineering, Technical University of Cluj-Napoca, Baritiu 25, 400027 Cluj-Napoca, Romania; nicoleta.cobarzan@ccm.utcluj.ro

**Keywords:** photothermal techniques, photopyroelectric calorimetry, thermal effusivity, porous solids, building materials

## Abstract

A new photopyroelectric detection configuration is proposed in order to measure the thermal effusivity of porous solids. Compared with the previously reported detection scheme this configuration makes use of a transparent window in front of the pyroelectric sensor. In such a way, the heat losses by convection at the sensor’s irradiated surface are eliminated, and consequently, the conduction remains the only process responsible for the heat propagation in the whole detection cell. In the paper, the mathematical model for this new configuration is developed, with the main conclusion that the sample’s thermal effusivity can be finally obtained via a fitting procedure with only two fitting parameters (instead of three as previously reported); in such a way, the possible degeneracy of the results is eliminated. The suitability of the method is demonstrated with application on some porous building materials and cellulose-based pressed powders.

## 1. Introduction

During the last few decades, the photopyroelectric (PPE) technique has been shown to be a very suitable method for the thermal characterization of a large class of condensed matter materials [1,2,3,4]. The compatibility of the PPE technique with homogeneous materials with well-defined properties (liquids and liquid mixtures, various types of solids–metals, semiconductors, magnets, etc.), as well as with non-homogeneous products with complex structures (such as composite materials, foodstuffs, agricultural and biological products, or drugs) has been proven [5,6]. The main advantages of this technique were found to be its simplicity, high sensitivity, non-destructive character, and adaptability to practical restrictions imposed by the experimental requirements.

The best-suited samples for PPE investigations are the liquids because the thermal contact between a liquid sample and a solid sensor is perfect, and consequently, accurate results are expected [6]. When investigating solids, a coupling fluid is used between the sensor and sample in order to ensure good thermal contact [7,8]. However, the coupling fluid can be used only for non-porous solids because, for porous materials, any type of liquid or paste coupling fluid will penetrate the sample, and the obtained result will be false. The only alternative to investigate from a thermal point of view, such as porous samples by PPE method, is to replace the liquid/paste coupling fluid between the sensor and sample with a layer of air.

The first attempt to use air as a coupling fluid in a PPE experiment was proposed by Salazar et al. [1]. The method is based on the front detection configuration (FPPE). The method uses thin opaque pyroelectric sensors and leads to the direct measurement of the sample’s thermal effusivity by performing a fit of the experimental data (phase of the FPPE signal vs. modulation frequency of radiation) with three fitting parameters: sample’s thermal effusivity, heat losses by convection at the irradiated surface, and air gap thickness between sensor and sample. This method has been shown to be very useful for thermal investigation of a large class of solid materials. However, this configuration has a clear limitation: due to the multiparametric fitting procedure, sometimes the solutions of the fit can degenerate. Consequently, a lot of attention must be paid to reduce or eliminate the degeneracy of the results. The first method to control the degeneracy is to limit the variation range of two fitting parameters, namely (i) heat losses by convection and (ii) thickness of the air gap between the sensor and sample [9]. Generally, the air gap thickness between the porous sample and sensor can be estimated microscopically by analyzing the sample’s roughness. Concerning the heat losses by convection, it depends on the experimental setup, mainly on the design of the detection cell (the quantity of air in contact with the sensor’s irradiated surface), on the thickness of the sensor, and on the power and diameter of the laser beam. In conclusion, its estimation is more difficult, and its elimination as a parameter in the fitting procedure (if possible) seems to be an important step to guarantee the unicity of the result obtained for the sample’s thermal effusivity.

This is, in fact, the purpose of this paper: to improve Salazar’s method [1] by eliminating one fitting parameter (the heat losses by convection). This improvement is performed experimentally by a new design of the detection cell. The large quantity of air in front of the irradiated sensor is eliminated by inserting a front glass window. The remaining thin layer of air will act as a coupling fluid between the sensor and the transparent window, but no heat propagation by convection will take place. This change leads practically to a new configuration of the detection cell, and consequently, a new theoretical model must be elaborated. Some measurements on solids with known values of thermal effusivity will validate the new theoretical approach. Investigations on several porous building materials will prove the suitability of this new method in a field of increasing interest: heat transfer in building materials in an unsteady state regime. The thermal effusivity of some cellulose-based pressed powders, representing another example of porous solids, was also measured.

## 2. Theory and Mathematical Simulations

**Figure 1:**
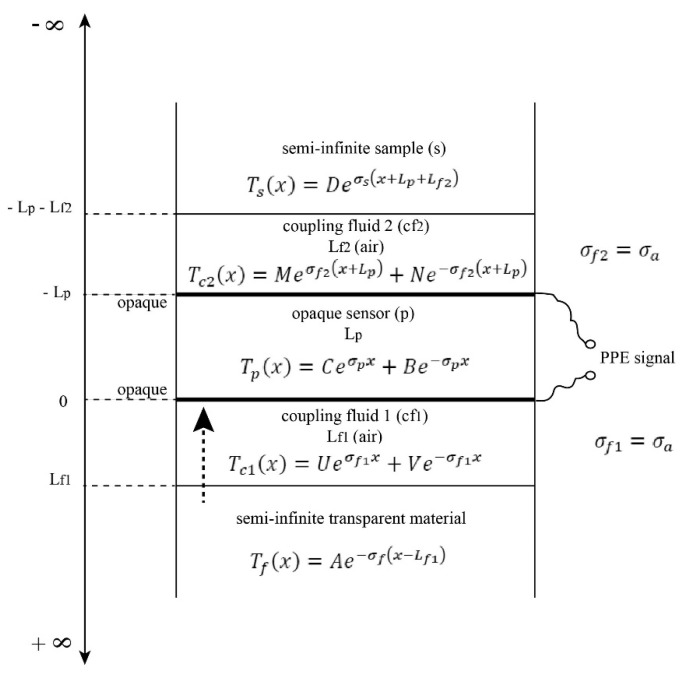
Schematic presentation of the detection cell.

The schematic view of the new proposed detection cell, together with the mathematical solutions for the temperature field in each layer of the detection cell, is displayed in Figure 1. The difference between this detection cell and the one proposed in Ref. [1] is the addition of the semi-infinite transparent material in front of the opaque sensor. From a theoretical point of view, the presence of this window means the addition of two new layers (coupling fluid *cf1*-air and the semi-infinite transparent material). Even if this detection cell is more complicated (compared with the one reported in [1]), it has the advantage that the only mechanism considered for heat propagation is heat conduction. It is also worth noting that (due to its transparency) the window in front of the sensor will bring no limitations concerning the experimental frequency range because the incident light will be absorbed by the same opaque surface of the sensor.

In order to obtain the PPE signal, one must solve the classical system of heat diffusion equations with particular conditions of continuity for temperature and heat flux at interfaces, as requested by the configuration described in Figure 1. The calculations are standard [3,4,5,6], and we will herein present only the particularities connected with the new configuration.

The average temperature across the pyroelectric sensor is given by

(1) $$\langle T_{p}(x)\rangle =\frac{1}{L_{p}}\overset{-L_{p}}{\underset{0}{\int }}\left(Ce^{\sigma_{p}x}+Be^{-\sigma_{p}x}\right)dx=\frac{1}{L_{p}\sigma_{p}}\left[C\left(e^{-\sigma_{p}L_{p}}-1\right)-B\left(e^{\sigma_{p}L_{p}}-1\right)\right]$$

where


(2) $$B=C\epsilon e^{-2\sigma_{p}L_{p}}$$

Consequently

(3) $$\langle T_{p}(x)\rangle ==\frac{C}{L_{p}\sigma_{p}}\left(e^{-\sigma_{p}L_{p}}-1\right)(1+\epsilon e^{-\sigma_{p}L_{p}})$$

with

(4) $$\varepsilon =\frac{e^{-2\sigma_{a}L_{f2}}\left(1+b_{pa}-b_{sa}b_{pa}-b_{sa}\right)-\left(1+b_{sa}-b_{pa}b_{sa}-b_{pa}\right)}{\left(1+b_{sa}+b_{pa}+b_{pa}b_{sa}\right)-e^{-2\sigma_{a}L_{f2}}\left(1-b_{pa}-b_{sa}+b_{sa}b_{pa}\right)}$$

In Equations (1)–(4), classical notations have been used: *b_ij_ = e_i_*/*e_j_* represents the effusivity ratio at the *ij* interface, *σ_j_ =* (1 *+ i*)*a_j_* where *a_j_ =* (*πf/α_j_*)^1/2^ represents the reciprocal of the thermal diffusion length in the *j* material. The subscripts *p*, *s*, *a* denote the pyroelectric sensor, sample, and air layers, respectively.

The quantity *C* can be obtained by using the relationships for flux and temperature continuity at *x =* 0 and *x = L_f_*_1_ interfaces. After some algebra and considering that the two coupling fluids are air (b_f1p_, b_f1s_ = 0), we obtain

(5) $$C=\frac{H}{K_{p}\sigma_{p}}\cdot \frac{1}{1-\epsilon \cdot e^{-2\sigma_{p}L_{p}}}$$

where *H* represents the light intensity absorbed by the irradiated sample’s surface at *x =* 0.

In order to eliminate the parameters connected with the experimental setup, a normalization measurement (without a sample) is necessary.

Finally, we will obtain for the normalized PPE signal the relationship:

(6) $$V_{n}=\frac{\langle T_{p}(x)\rangle }{{\langle T_{p}(x)\rangle }_{n}}=\frac{1+\epsilon \cdot e^{-\sigma_{p}L_{p}}}{1-\epsilon \cdot e^{-2\sigma_{p}L_{p}}}\cdot \frac{1-\epsilon^{\prime }e^{-2\sigma_{p}L_{p}}}{1+\epsilon^{\prime }e^{-\sigma_{p}L_{p}}}$$

where


(7) $$\epsilon^{\prime }=\frac{b_{pa}-1}{b_{pa}+1}$$

Equation (6) indicates that the normalized PPE signal is, as expected, a complex quantity. Both amplitude and phase of the signal can be used in order to obtain the sample’s thermal effusivity via a fitting procedure with only two fitting parameters: the sample’s thermal effusivity, *e_s_,* and the coupling fluid’s thickness *L_f_*_2_. It is important to mention that the final result does not depend on the thermal and geometrical parameters of the semi-infinite material in front of the sensor and the coupling fluid *c_f_*_1_. During the experiment, we will use the phase of the normalized PPE signal as a source of information because, as it is wellknown, it is less noisy than the amplitude [5,6].

**Figure 2:**
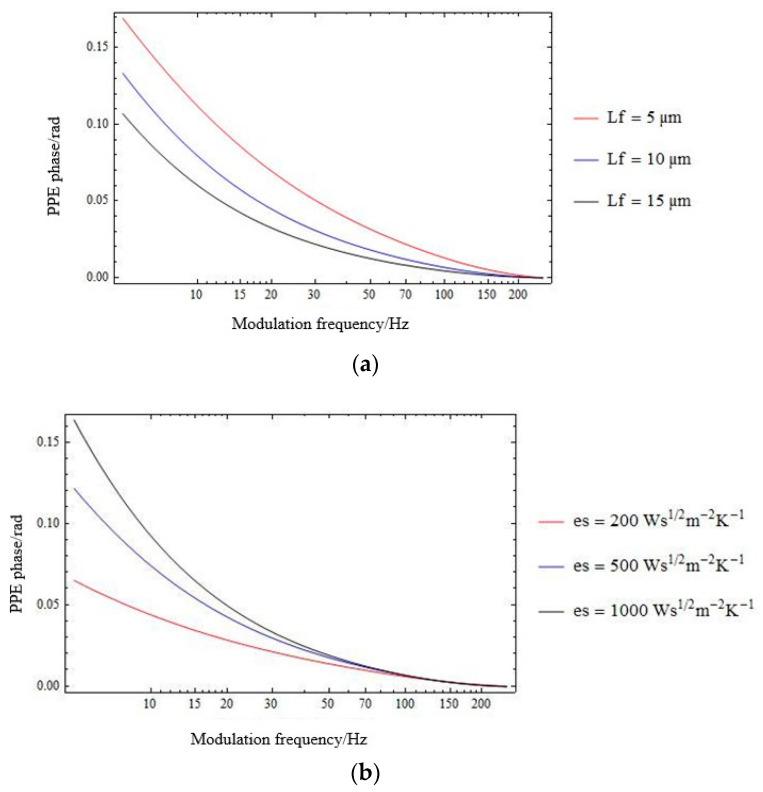
Mathematical simulations for the phase of the PPE signal as described by Equation (6), for (**a**) *e_s_* = 500 Ws^½^m^−2^K^−1^ and air gap thickness *L_f_*_2_ = 5 µm; 10 µm; 15 µm; and (**b**) *L_f_*_2_ = 10 µm and *e_s_* = 200 Ws^½^m^−2^K^−1^; 500 Ws^½^m^−2^K^−1^; 1000 Ws^½^m^−2^K^−1^.

Figure 2a,b contains mathematical simulations for the phase of the PPE signal as described by Equation (6) for some typical effusivity (*e_s_*) (Figure 2a) and coupling fluid’s thickness (*L_f2_*) (Figure 2b) values.

## 3. Experimental

The experimental setup was typical for PPE experiments [5,6,10]. The radiation source was a 100 mW YAG laser. The modulation frequency range (5–250 Hz) and the diameter of the laser beam (8 mm) were selected to assure the approximation for one-directional propagation of heat through the detection cell. The pyroelectric sensor was 15 × 15 mm^2^ area LiTaO_3_ single crystals (400 µm and 100 µm thick and provided with Cr-Au electrodes on both sides). The semi-infinite transparent window in front of the sensor was a 5 mm thick quartz glass, transparent in both visible and near-infrared (NIR). In such a way, any additional heat source generated in the window by the absorption of the IR radiation emitted by the heated sensor’s surface is eliminated. An SR-830 lock-in amplifier was used for data acquisition, and a PC was used for data processing. The measurement consisted, in fact, of a scan of the phase of the FPPE signal as a function of the modulation frequency. The information is collected from the phase of the signal (and not from the amplitude) because, as mentioned before, the phase is less noisy than the amplitude of the signal. All the measurements have been performed at room temperature, and the S/N ratio for all the measurements was better than 100. The uncertainties in the thermal effusivities were calculated from the RMS deviation of the measured data compared with the fitting curve. They are generally in the range of 2.5–3%. The uncertainties in La were always lower than 1 µm.

As samples, we selected firstly two “classical” samples with rather low values for thermal effusivity (Teflon and wood) in order to support the validity of the method. As an application, some porous building materials of large interest in construction (clay bricks, autoclaved aerated concrete, limestone), encompassing a rather large scale of thermal effusivity values (100–1200 Ws^1/2^m^−2^K^−1^), have been investigated. The geometry of the building materials used in experiments was 1 cm^2^ flat surface in contact with the sensor and with a thickness larger than 5 mm.

Fired clay bricks are porous materials [11] whose thermal characteristics [12] are influenced by volume, size, distribution of pores, materials anisotropy [13], firing temperature, and chemical and mineralogical composition of raw materials. Depending on the percentage, type, and size of the pore-forming agent, the pores have irregular shapes, are elongated, and are orientated parallel to the extrusion direction. In the case of autoclaved aerated concrete (AAC), the pores are rounded or spherical [12,13], with more uniform distribution in the sample mass compared with clay bricks. 

Usually, the pores of fired clay bricks and AAC are less than 3 mm in diameter [11,14]. They can be closed or open and interconnected, which makes them sensitive to water absorption. Consequently, the measurement of these materials by using the standard PPE method (using coupling fluid) is clearly not recommended.

The commercially available powders of pure Lignin-L (alkali, Sigma Aldrich, Burligton, MA, USA) and pure Methylcellulose–M (Sigma Aldrich) are used for the preparation of cellulose-pressed powders. Equal amounts of lignin and methyl cellulose are taken and ground using an agate mortar for 30 min to obtain a cellulose-mixed lignin sample (ML). These powders are pressed into pellets (13 mm in diameter) by giving a load of 10 Tons for 5 min using Specac manual hydraulic press (using 13 mm Evacuable Pellet Die, Orpington, UK). The thickness of L, M, and ML samples measured using digital vernier (Beta 1651 L200/DGT) are 2.40 mm, 3.82 mm, and 5.44 mm, respectively. The camphor soot (ca-soot) powder obtained by the incomplete combustion of camphor tablets and diesel soot (die-soot) powder collected from exhaust pipes of the internal combustion engines is also pressed into pellets of 13 mm diameter by giving a load of 10 Tons for 5 min in the hydraulic press. The thickness of the pellets of camphor soot and diesel soot is 2.17 mm and 1.51 mm, respectively. The soot samples are rich in various allotropes of carbon like graphene, fullerenes, amorphous carbon, graphite, and multi-walled and single-walled carbon nanotubes [15,16,17]. Their size is found to be in the range of 20–60 nm [15,17].

## 4. Results

**Figure 3:**
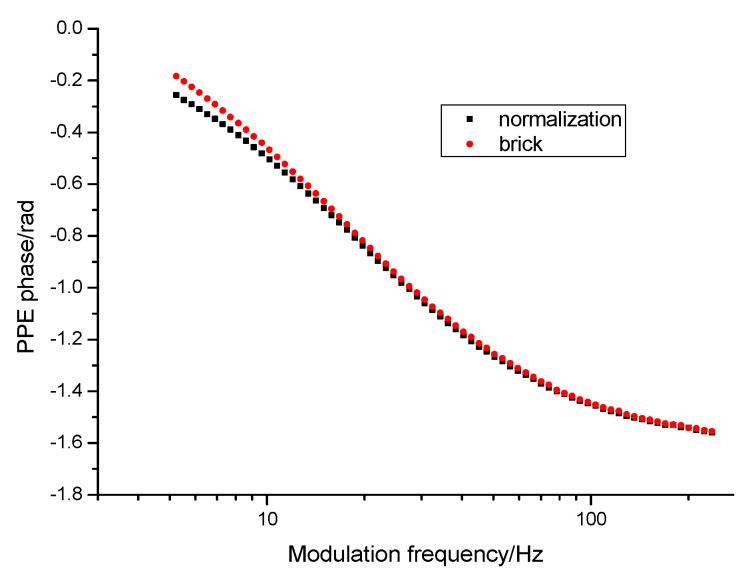
Frequency behavior of the phase of the PPE signal as obtained for a brick sample.

Figure 3 displays a typical measurement performed for a brick sample over a large frequency range. The behavior of the phase of the FPPE signal for the sample (brick) and for normalization are displayed. The behavior of the normalized phase is obtained by the difference of the two curves.

**Figure 4:**
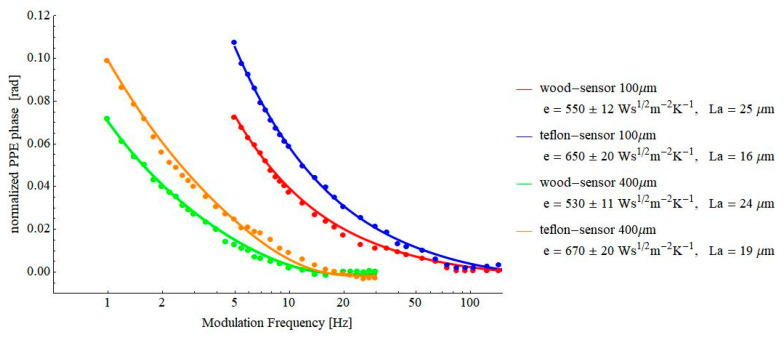
Behavior of the normalized FPPE phase as a function of the modulation frequency for wood and Teflon samples and for two thicknesses of the pyroelectric sensor, 400µm and 100 µm, respectively.

In fact, experimentally, one of the most important decisions is to select the thickness of the pyroelectric sensor used in the experiment. Figure 4 displays the results obtained for two “classical” samples, Teflon and wood, and for two thicknesses of the pyroelectric sensor.

The values of the thermal effusivity of wood and Teflon, obtained for the two sensor’s thicknesses, are in good agreement, and they are also in agreement with literature data (600–740 Ws^½^m^−2^K^−1^for Teflon [5,18,19] and 470–679 Ws^½^m^−2^K^−1^ for wood [18,19], respectively). However, for further investigations, we selected the 100 µm thick LiTaO_3_ sensor mainly because it allows measurements in a higher frequency range. Sometimes, samples, such as pressed powders, cannot be obtained with large enough thicknesses, and consequently, the theoretical request of a thermally thick sample cannot be satisfied at low frequencies.

**Figure 5:**
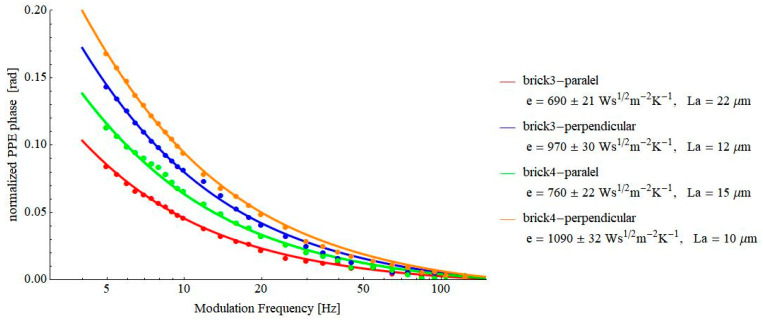
Frequency behavior of the normalized phase of the FPPE signal as obtained for two bricks, detected in a direction parallel and perpendicular to the extrusion direction.

**Figure 6:**
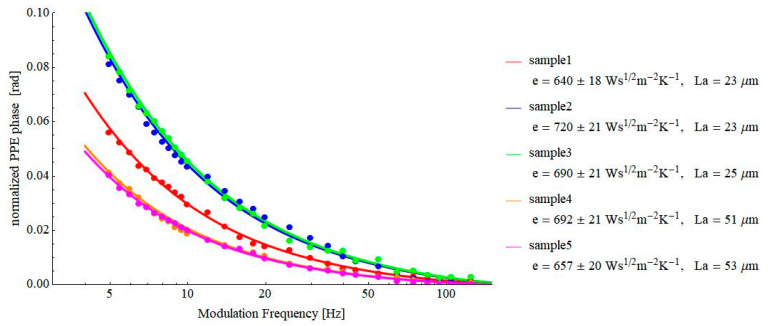
Frequency behavior of the normalized phase of the FPPE signal as obtained for several bricks, all detected in a direction parallel with the extrusion direction.

The results obtained for some brick samples are displayed in Figure 5 and Figure 6.

The results presented in Figure 5 are in agreement with the literature data (510–1079 Ws^½^m^−2^K^−1^ [19,20,21,22]. At the same time, there is a clear difference between the values of the thermal effusivity along or perpendicular to the extrusion direction.

One of the main problems when using the PPE method for investigating building materials is the non-uniformity of the investigated materials (which could be measured as a whole with other methods—the hot plate method, for example). Bricks are generally non-uniform inhomogeneous materials, and the application of a method such as PPE, which is sensitive just to the “local” properties of the material in contact with the sensor, can raise some questions of validity. This is why we investigated several pieces (subsamples) of a brick to check for the degree of inhomogeneity. The results are presented in Figure 6.

As results from Figure 6, the obtained average thermal effusivity is 680 ± 32 Ws^½^m^−2^K^−1^, which indicates that the thermal effusivity of the brick can be determined with a precision expressed as reproducibility of 13% at a 95% confidence level. This is despite the fact that the measurements have to be performed on different parts of a relatively inhomogeneous material. The standard deviations of effusivities determined for each brick piece are about 3%. For the hot plate method, reports indicate reproducibilities of 5.5% (95% confidence level). The presented level of precision supports the PPE technique as a suitable test method for porous samples, including relatively inhomogeneous samples such as brick.

**Figure 7:**
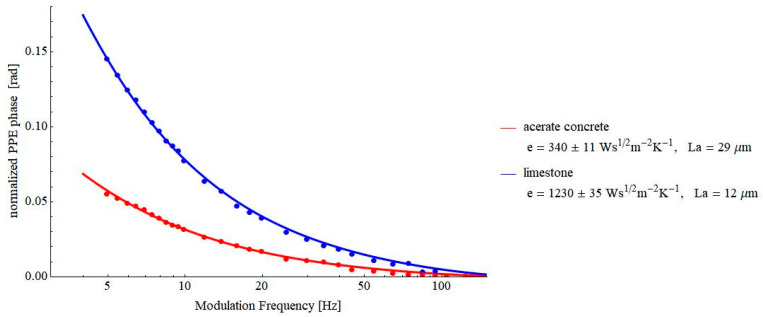
Frequency behavior of the normalized phase of the FPPE signal as obtained for AAC and limestone.

Figure 7 contains the results obtained on other building materials of interest: autoclaved aerated concrete and limestone. As mentioned above, autoclaved aerated concrete (AAC) is a building material used in masonry or framed buildings due to its good thermal performance. It has large pores, uniformly distributed in the mass of the sample [22,23,24,25]. Due to the large quantity of air inside the material, the value of the thermal effusivity is rather low, as resulted from Figure 7. The obtained result is in good agreement with the literature data (361 Ws^½^m^−2^K^−1^ [19]).

Limestone was selected for investigations not only because it is a building material of interest but also because it can be easily processed as a sample with various geometries. It is well-known that the classical method used by specialists for thermal investigations of building materials is the hot-plate method. This method gives the value of the thermal conductivity of a sample in a stationary thermal regime, but it requests some typical geometry of the investigated sample. Unfortunately, often, the samples under investigation already have a pre-elaborated geometry that does not fit with the requests of the hot-plate equipment. In such cases, a method such as PPE, which is independent of the sample’s geometry, can be a solution. Going back to limestone, it was selected as a sample in our experiments because it can be processed with geometries requested by both PPE and classical hot-plate methods, allowing for a comparison of the results, if necessary. The result for the thermal effusivity obtained by the PPE method is in agreement with the literature data (1182 Ws^½^m^−2^K^−1^ [19]).

**Figure 8:**
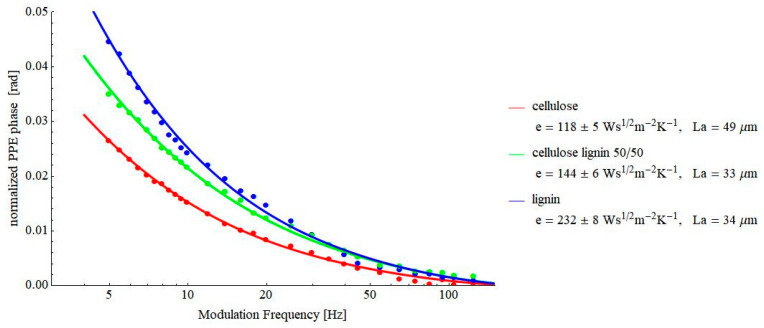
Frequency behavior of the normalized phase of the FPPE signal as obtained for cellulose–lignin mixtures.

**Figure 9:**
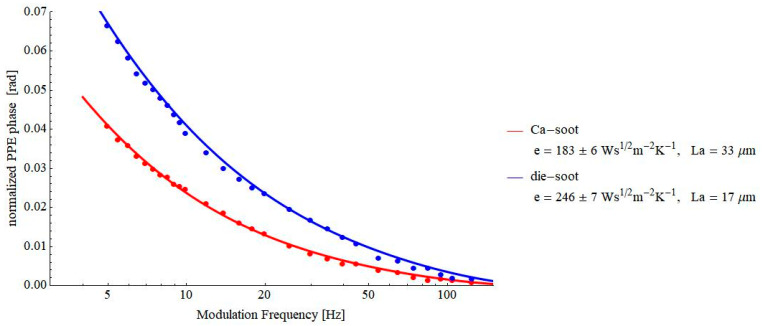
Frequency behavior of the normalized phase of the FPPE signal as obtained for soot-based samples.

Figure 8 and Figure 9 contain the results obtained on some pressed powders (cellulose–lignin mixtures and soot-based materials, respectively). These materials were selected for investigations because they constitute another example of porous solids. At the same time, a lot of air is incorporated into their structure, and consequently, the value of the thermal effusivity is rather low, testing the limits of the proposed PPE method. As a result of Figure 7, Figure 8 and Figure 9, values of thermal effusivity in the range of 118–246 Ws^½^m^−2^K^−1^ have been obtained. As a comparison, Sanja et al. reported values of thermal effusivity of cellulose knitted fabrics in the range 80–120 Ws^½^m^−2^K^−1^ [23], but, of course, these values depend on the granulation of powders, the pressure used for obtaining the pills, etc.

## 5. Conclusions

In the paper, a new photopyroelectric detection configuration is proposed in order to measure the thermal effusivity of porous solids. Compared with the previously reported detection configurations [1], this one eliminates the heat losses by convection at the sensor’s irradiated surface by making use of a transparent window in front of the pyroelectric sensor. The mathematical model developed in the paper for this new configuration demonstrates that the sample’s thermal effusivity can be obtained via a fitting procedure with only two fitting parameters (instead of three as previously reported): the sample’s thermal effusivity and the air gap thickness between the sensor and sample. Considering that the air gap thickness between the sensor and sample can be estimated microscopically (by measuring the sample’s surface roughness), it seems that the possible degeneracy of the results is eliminated. 

As predicted by theory, the performed experiments confirm that the sensitivity of the method increases with decreasing the thicknesses of the pyroelectric sensor and air gap between sensor and sample. As expected, the sensitivity of the method increases with increasing the sample’s thermal effusivity value. However, the method can be used for investigations of samples with low thermal effusivities (140 Ws^½^m^−2^K^−1^—in the experiments with pressed powders).

The suitability of the method is demonstrated with application on some porous building materials and some cellulose-based pressed powders. Concerning the selection of this class of (building) materials, it is well-known that accurate estimation of thermophysical parameters of building materials is critical for the evaluation and improvement of energy efficiency in the building sector. Unfortunately, until now, the building materials characterization was mainly limited to the study of the thermal conductivity and the energy performance of buildings in a steady-state regime. Thermal effusivity used for the evaluation of heat transfer in an unsteady-state regime has received less attention so far. The thermal regime of a building can be considered for a limited time interval as stationary, but for 24 h, with significant temperature variations in a day/night cycle, the conditions are non-stationary. Thermal effusivity is an essential parameter for the evaluation of the thermal inertia of materials and optimizing the structure of envelope members in order to increase thermal comfort in buildings by avoiding overheating and reducing the energy demand for heating and cooling [25,26]. This is why its correct and accurate measurement becomes very important.

The results for the investigated building materials samples are in good agreement with the available literature data. Similarly, the results obtained for pressed powders proved that this type of material can also be investigated from a thermal point of view by the PPE technique in the proposed configuration. Due to the fact that the value of the thermal effusivity of these materials is rather low (they contain a lot of air in their structure), these pressed powders constitute test materials for the lower limit of the thermal effusivity that can be investigated by the proposed method. It resulted that values around 100 Ws^½^m^−2^K^−1^ can be correctly measured with enough accuracy. Unfortunately, no comparison of the results obtained by PPE on these pressed powders with literature data is possible because the value of the thermal effusivity depends on the granulation and pressure used during the sample processing.

Finally, we want to stress once more the statement that porous solids belong to a class of materials that are very difficult to be investigated by PPE. To our knowledge, porous solids represent the only type of materials belonging to condensed matter that were not taken into consideration (until recently) as potential samples for PPE calorimetric investigations [27,28,29]; this is due to the fact that it is practically impossible to perform a good thermal contact between two solids when one is porous. Consequently, the alternative method proposed in this paper for their thermal characterization completes the area of application of the PPE method. However, even this method can be improved; work is in progress with experiments in a new PPE configuration that allows the direct measurement of two thermal parameters (thermal effusivity and diffusivity) of porous solids by using fitting procedures involving only one fitting parameter. Such a configuration allows a *complete* thermal characterization (all static and dynamic thermal parameters are obtained) of the porous material and, at the same time, constitutes a warranty for the *unicity* of the solution.

## Data Availability

No new data were created.

## References

[1] Salazar A., Oleaga A., Mendioroz A., Apiñaniz E. (2018). Thermal effusivity measurements of thermal insulators using the photopyroelctrictechnique in the front configuration. Measurement.

[2] Zammit U., Mercuri F., Paoloni S., Marinelli M., Pizzoferrato R. (2015). Simultaneous absolute measurements of the thermal diffusivity and the thermal effusivity in solids and liquids using phtopyroelectric calorimetry. J. Appl. Phys..

[3] Mandelis A., Zver M.M. (1985). Theory of photopyroelectric spectroscopy of solids. J. Appl. Phys..

[4] Chirtoc M., Mihailescu G. (1989). Theory of the photopyroelectric method for investigations of optical and thermal materials properties. Phys. Rev. B.

[5] Dadarlat D. (2012). Contact and non-contact photothermal calorimetry for investigation of condensed matter. J. Therm. Analysis Calor..

[6] Dadarlat D. (2009). Photopyroelectric calorimetry of liquids; recent development and applications. Laser Phys..

[7] Salazar A. (2003). On the influence of the coupling fluid in photopyroelectric measurements. Rev. Sci. Instrum..

[8] Salazar A., Oleaga A. (2012). Overcoming the influence of the coupling fluid in photopyroelectric measurements of solid samples. Rev. Sci. Instrum..

[9] Tripon C., Dadarlat D., Kovacs K., Tosa V.P., Franko M. (2020). Thermal effusivity investigations of solid thermoelectrics using the front pyroelectric detection. Int. J. Thermophys..

[10] Dadarlat D., Bicanic D., Visser H., Mercuri F., Frandas A. (1995). Photopyroelectric Method for Determination of Thermophysical Parameters and Detection of Phase Transitions in Fatty Acids and Triglycerides. Part I: Principles, Theory and Instrumentational Concepts. J. Am. Oil Chem. Soc..

[11] Stryszewska T., Kańka S. (2019). Forms of Damage of Bricks Subjected to Cyclic Freezing and Thawing in Actual Conditions. Materials.

[12] Bories C., Borredon M.E., Vedrenne E., Vilarem G. (2014). Development of eco-friendly porous fired clay bricks using pore-forming agents: A review. J. Environ. Manag..

[13] Maillard P., Aubert J.P. (2014). Effects of the anisotropy of extruded earth bricks on their hygrothermal properties. Construct. Build. Mater..

[14] Schober G. (2011). Porosity in autoclaved aerated concrete (AAC): A review on pore structure, types of porosity, measurement methods and effects of porosity on properties (Special issue). Cement WapnoBeton.

[15] Swapna M.S., Sankararaman S. (2018). Blue Light Emitting Diesel Soot for Photonic Applications. Mater. Res. Express.

[16] Swapna M.S., Sankararaman S. (2019). Thermal Induced Order Fluctuations in Carbon Nanosystem with Carbon Nanotubes. Nano-Struct. Nano-Objects.

[17] Swapna M.S., Saritha Devi H.V., Sankararaman S. (2018). Camphor Soot: A Tunable Light Emitter. Appl. Phys. A.

[18] Mandelis A. (1992). Principles and Perspective of Photothermal and Photoacoustic Phenomena.

[19] Thermtest Materials Database. https://thermtest.com/thermal-resources/materials-database.

[20] Raut A.N., Gomez C.P. (2017). Performance Evaluation of newly developed sustainable blocks for affordable housing in Malaysia. MATEC Web Conf..

[21] Laaroussi N., Cherki A., Garoum M., Khabbazi A., Feiz A. (2013). Thermal properties of a sample prepared using mixtures of clay bricks. Energy Procedia.

[22] Lamrani M., Mansour M., Laaroussi N., Khalfaoui M. (2019). Thermal study of clay bricks reinforced by three ecological materials in south of Morocco. Energy Procedia.

[23] Pavlović S.S., Stanković S.B., Popović D.M., Poparić G.B. (2014). Transient Thermal Response of Textile Fabrics Made of Natural and Regenerated Cellulose Fibers. Polym. Test..

[24] Blaine R.L. (2018). In search of thermal effusivity reference materials. J. Therm. Anal. Calorim..

[25] Aste N., Leonforte F., Manfren M., Mazzon M. (2015). Thermal inertia and energy efficiency—Parametric simulation assessment on a calibrated case study. J. App. Energy.

[26] Kuczyński T., Staszczuk A. (2020). Experimental study of the influence of thermal mass on thermal comfort and cooling energy demand in residential buildings. Energy.

[27] Marinelli M., Mercuri F. (2000). Effects of fluctuations in the orientational order parameter in the cyanobiphenyl (nCB) homologous series. Phys. Rev. E.

[28] Zammit U., Marinelli M., Mercuri F., Paoloni S., Scudieri F. (2011). Effect of quenched disorder on the RI-RV, R II-RI, and liquid-RII rotator phase transitions in alkanes. J. Phys. Chem. B.

[29] Dadarlat D., Tripon C., White I.R., Korte D. (2022). Photopyroelectric spectroscopy and calorimetry. J. Appl. Phys..

